# Diabetes status modifies the long-term effect of lipoprotein-associated phospholipase A2 on major coronary events

**DOI:** 10.1007/s00125-021-05574-5

**Published:** 2021-09-25

**Authors:** Moneeza K. Siddiqui, Gillian Smith, Pamela St Jean, Adem Y. Dawed, Samira Bell, Enrique Soto-Pedre, Gwen Kennedy, Fiona Carr, Lars Wallentin, Harvey White, Colin H. Macphee, Dawn Waterworth, Colin N. A. Palmer

**Affiliations:** 1 Division of Population Health and Genomics, Ninewells Hospital and Medical School, University of Dundee, Dundee, UK; 2 PAREXEL International, Durham, NC USA; 3 Department of Medical Sciences, Cardiology, Uppsala University, Uppsala, Sweden; 4 Uppsala Clinical Research Center, Uppsala University, Uppsala, Sweden; 5 Green Lane Cardiovascular Service, Auckland City Hospital and University of Auckland, Auckland, New Zealand; 6 Novel Human Genetics, GlaxoSmithKline, Collegeville, PA USA; 7 Department of Genetics, GlaxoSmithKline Medicines Research Centre, Philadelphia, PA USA

**Keywords:** Clinical diabetes, Epidemiology, Lipids, Lipoproteins, Major coronary events, Precision medicine, Type 2 diabetes

## Abstract

**Aims/hypothesis:**

Lipoprotein-associated phospholipase A2 (Lp-PLA2) activity has an independent prognostic association with major coronary events (MCE). However, no study has investigated whether type 2 diabetes status modifies the effect of Lp-PLA2 activity or inhibition on the risk of MCE. We investigate the interaction between diabetes status and Lp-PLA2 activity with risk of MCE. Subsequently, we test the resulting hypothesis that diabetes status will play a role in modifying the efficacy of an Lp-PLA2 inhibitor.

**Methods:**

A retrospective cohort study design was utilised in two study populations. Discovery analyses were performed in the Genetics of Diabetes Audit and Research in Tayside Scotland (GoDARTS) cohort based in Scotland, UK. Participants were categorised by type 2 diabetes control status: poorly controlled (HbA_1c_ ≥ 48 mmol/mol or ≥6.5%) and well-controlled (HbA_1c_ < 48 mmol/mol or <6.5%) diabetes (*n* = 7420). In a secondary analysis of the Stabilization of Atherosclerotic Plaque by Initiation of Darapladib Therapy (STABILITY) trial of Lp-PLA2 inhibitor (darapladib) efficacy, 15,828 participants were stratified post hoc by type 2 diabetes diagnosis status (diabetes or no diabetes) at time of recruitment. Lp-PLA2 activity was then divided into population-specific quartiles. MCE were determined from linked medical records in GoDARTS and trial records in STABILITY. First, the interaction between diabetes control status and Lp-PLA2 activity on the outcome of MCE was explored in GoDARTS. The effect was replicated in the placebo arm of STABILITY. The effect of Lp-PLA2 on MCE was then examined in models stratified by diabetes status. This helped determine participants at higher risk. Finally, the effect of Lp-PLA2 inhibition was assessed in STABILITY in the higher risk group. Cox proportional hazards models adjusted for confounders were used to assess associations.

**Results:**

In GoDARTS, a significant interaction between increased Lp-PLA2 activity (continuous and quartile divided) and diabetes control status was observed in the prediction of MCE (*p* < 0.0001). These effects were replicated in the placebo arm of STABILITY (*p* < 0.0001). In GoDARTS, stratified analyses showed that, among individuals with poorly controlled diabetes, the hazards of MCE for those with high (Q4) Lp-PLA2 activity was 1.19 compared with individuals with lower (Q1–3) Lp-PLA2 activity (95% CI 1.11, 1.38; *p* < 0.0001) and 1.35 (95% CI 1.16, 1.57; *p* < 0.0001) when compared with those with the lowest activity (Q1). Those in the higher risk group were identified as individuals with the highest Lp-PLA2 activity (Q4) and poorly controlled diabetes or diabetes. Based on these observations in untreated populations, we hypothesised that the Lp-PLA2 inhibitor would have more benefit in this higher risk group. In this risk group, Lp-PLA2 inhibitor use was associated with a 33% reduction in MCE compared with placebo (HR 0.67 [95% CI 0.50, 0.90]; *p* = 0.008). In contrast, Lp-PLA2 inhibitor showed no efficacy in individuals with low activity, regardless of diabetes status, or among those with no baseline diabetes and high Lp-PLA2 activity.

**Conclusions/interpretation:**

These results support the hypothesis that diabetes status modifies the association between Lp-PLA2 activity and MCE. These results suggest that cardiovascular morbidity and mortality associated with Lp-PLA2 activity is especially important in patients with type 2 diabetes, particularly those with worse glycaemic control. Further investigation of the effects of Lp-PLA2 inhibition in diabetes appears warranted.

**Data availability:**

STABILITY trial data are available from clinicaltrials.gov repository through the GlaxoSmithKline clinical study register https://clinicaltrials.gov/ct2/show/NCT00799903. GoDARTS datasets generated during and/or analysed during the current study are available following request to the GoDARTS Access Managements Group https://godarts.org/scientific-community/.

**Graphical abstract:**

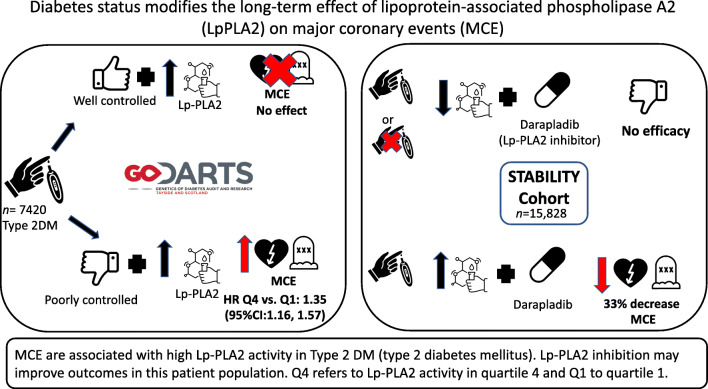

**Supplementary Information:**

The online version of this article (10.1007/s00125-021-05574-5) contains peer-reviewed but unedited supplementary material.

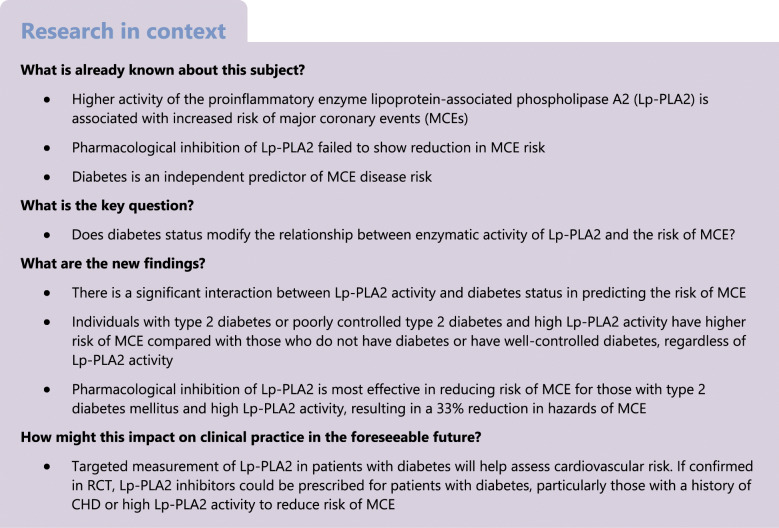



## Introduction

The prognostic association of lipoprotein-associated phospholipase A2 (Lp-PLA2) with cardiovascular health and outcomes, independent of traditional risk factors such as LDL-cholesterol (LDL-c) was first established by the West of Scotland Coronary Prevention Study over 19 years ago [1]. Since then, several studies have replicated this finding, and Lp-PLA2 was identified as a viable drug target for the prevention of coronary events [2, 3].

Coronary events are caused by the rupture of atherosclerotic plaques, and a critical step in the formation of atherosclerotic coronary lesions is the accumulation and oxidation of LDL particles. The vast majority of Lp-PLA2 binds to the LDL-c particle while circulating in the blood stream, and a smaller proportion to the HDL-c particle [4]. In vitro studies have shown that increased LDL-c results in upregulation of Lp-PLA2, suggesting their regulation in plasma is closely linked even if their biosynthesis is independent [5]. Therefore, hypothetically, inhibiting the enzyme should have a cardioprotective effect independent of cholesterol-lowering therapies.

However, trials with the first Lp-PLA2 inhibitor, darapladib, were unsuccessful. The Stabilization of Atherosclerotic Plaque by Initiation of Darapladib Therapy (STABILITY) trial in individuals with chronic heart disease did not meet its primary outcome of major adverse cardiovascular events (MACE) [6], though darapladib (SB-480848) did reduce the risk of the secondary endpoint of major coronary event (MCE) by 10% (HR 0.90 [95% CI 0.82, 1.00]) [6]. The difference in these endpoints is the inclusion of ischaemic strokes in MACE. Observational studies have failed to find evidence of a relationship between Lp-PLA2 activity and ischaemic stroke, summarised in the meta-analysis by Thompson et al [2]. The Stabilization of Plaque Using Darapladib-Thrombolysis in Myocardial Infarction 52 (SOLID-TIMI 52) trial found that darapladib did not reduce the risk of MCE in individuals who had experienced an acute coronary event [7]. Efforts to progress the drug for clinical use were therefore discontinued. Nevertheless, Lp-PLA2 remains a significant and, crucially, independent risk factor for ischaemic events, with an additional hypothesised role in the development of diabetic retinopathy [8]. However, at present it is not part of any clinical risk scores for ischaemic events.

Diabetes is a well-established risk factor for CVD. However, the interaction of type 2 diabetes status and Lp-PLA2 activity in determining cardiovascular risk has never been examined in humans. In preclinical data, a reduction in coronary atherosclerosis with darapladib treatment in a type 2 diabetes porcine model of atherosclerosis was reported [3]. The *PLA2G7* gene on chromosome 6 encodes the protein Lp-PLA2. It has recently been demonstrated that adipose tissue from individuals with type 2 diabetes has higher levels of *PLA2G7* gene expression compared with that observed in individuals without diabetes [5]. This suggests a potential interaction could exist between diabetes status and Lp-PLA2 activity. The inflammatory pathways associated with high Lp-PLA2 activity could be modified by glycaemic control, which also has a known relationship with inflammation [9–11]. Thus Lp-PLA2 and dysglycaemia could synergistically increase the atherosclerotic plaque instability. This study examines if diabetes status modifies the relationship between Lp-PLA2 activity and cardiovascular outcomes in a large retrospective cohort study. The hypothesis generated from the observations was tested in a post hoc analysis of the STABILITY trial.

## Methods

### Study design

Discovery analyses were performed in the Genetics of Diabetes Audit and Research in Tayside Scotland (GoDARTS) cohort. GoDARTS is an observational cohort study with record-linked medical and prescribing history for 16,838 participants, who consented to participate in the study [12]. Ethical approval for the study was provided by the Tayside Medical Ethics Committee (REF:053/04) and the study has been carried out in accordance with the Declaration of Helsinki. Lp-PLA2 activity was measured in individuals with type 2 diabetes (*n* = 7420) and the study was performed in this group. The STABILITY trial was approved by national regulatory authorities and by local ethics committees or institutional review boards according to relevant local regulations. For this study, replication was sought through a secondary analysis of the STABILITY trial (registration number: NCT00799903), where 15,828 individuals were randomised to receive either Lp-PLA2 inhibitor (darapladib) or placebo. The effect of Lp-PLA2 activity on MCE in the prognostic/placebo-treated arm of the trial was examined. The effect of Lp-PLA2 inhibition (darapladib therapy) on MCE was examined in the full trial population. Retrospective cohort analyses were performed in both the GoDARTS cohort and in the STABILITY trial.

The study details three key results. First, we assess the interaction between Lp-PLA2 activity and diabetes status in predicting the risk of MCE. Second, we stratify the association of Lp-PLA2 with MCE risk by diabetes status. This stratification allows us to determine the higher risk group. Third, we test the efficacy of Lp-PLA2 inhibition in this higher risk group. The interaction and stratification results are demonstrated in GoDARTS and the placebo-treated arm of STABILITY, while the effect of Lp-PLA2 inhibition is demonstrated in the STABILITY trial population.

### Measurement and use of Lp-PLA2 activity

There are two FDA-approved platforms for the detection of Lp-PLA2 in sera or plasmas; one that measures enzymatic activity and another that measures mass of enzyme. The current and improved enzymatic activity assay (diaDexus PLAC test for Lp-PLA2 Activity; Diazyme, San Francisco, CA, USA) was used for the first time in the two large cardiovascular outcome trials with Lp-PLA2 inhibitor, darapladib (e.g. STABILITY) [13]. Measuring enzymatic activity is the preferred platform since there are recognised issues with the mass assay [14, 15]. Importantly, the same enzymatic activity assay was used for the analysis of Lp-PLA2 in GoDARTS [8] as was used for STABILITY. In both studies, Lp-PLA2 activity was measured from serum samples obtained at the time of recruitment [8].

Consistent with previous investigations of Lp-PLA2 activity, quartiles of Lp-PLA2 activity were generated for each study population [1, 8, 13]. Preliminary analyses of interaction with diabetes status were demonstrated using both linear and quartile transformed Lp-PLA2 activity.

### Diabetes status

In GoDARTS, HbA_1c_ levels were measured at recruitment, and Lp-PLA2 activity was measured from samples collected at recruitment. The WHO criteria for the diagnosis of type 2 diabetes recommends the use of HbA_1c_ 48 mmol/mol (6.5%) as a threshold for classification of diabetes [16]. We used this threshold in the study population to categorise the diabetes control status for participants into well-controlled (HbA_1c_ < 48 mmol/mol, *n* = 1979) and poorly controlled diabetes (HbA_1c_ ≥ 48 mmol/mol, *n* = 5441). In STABILITY, diabetes status at recruitment was recorded as type 2 diabetes requiring pharmacotherapy. HbA_1c_ was measured only in individuals with diabetes. Since STABILITY has the added comparison group of drug effect, interactions and stratifications were performed using diabetes status at recruitment. This approach allowed us to use the full trial population and enhance clinical applicability. All analyses in STABILITY were performed in the intent-to-treat population. Therefore, in GoDARTS, diabetes status refers to diabetes control status and in STABILITY, it refers to the presence or absence of type 2 diabetes diagnosis.

**Fig. 1:**
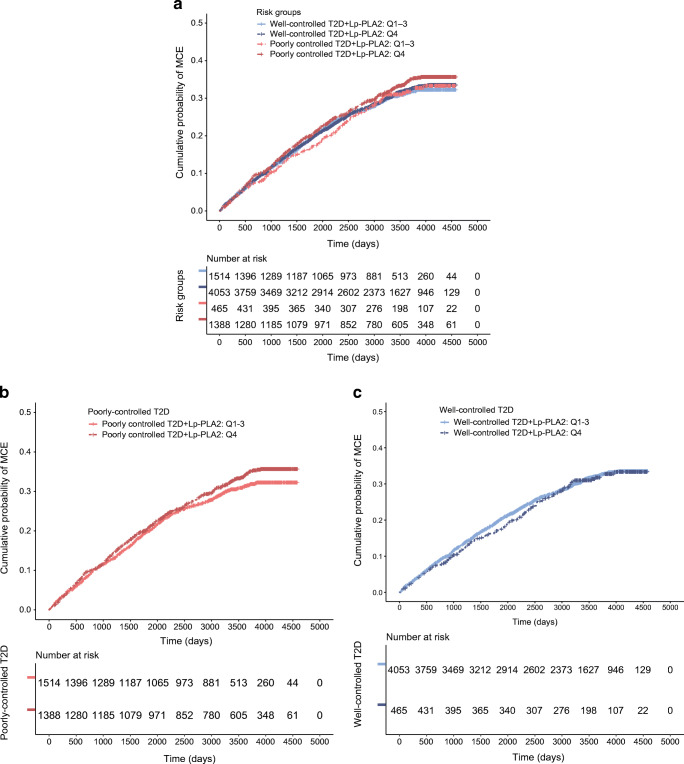
GoDARTS study: demonstration of all risk groups in GoDARTS. High vs lower Lp-PLA2 activity (Q4 vs Q1–3) and diabetes control status. (**a**) The association between diabetes control status and high vs low Lp-PLA2 activity was significant (*z* = 3.80, *p* < 0.0001, HR 1.07 [95% CI 1.04, 1.13]). (**b**) Stratified effect among participants with poorly controlled diabetes. Lp-PLA2 activity in the highest quartile (Q4) compared with activity in Q1–3 was associated with 1.19 times the hazards of MCE (95% CI 1.07, 1.33) *p* < 0.001. (**c**) Stratified effect among participants with well-controlled diabetes, no significant difference in hazards of MCE for those with Lp-PLA2 activity in the highest quartile compared with lower quartiles (HR 1.04 [95% CI 0.86, 1.27]) *p* = 0.69. Full model in Table 2. T2D, type 2 diabetes

A flow chart explaining the study populations is provided in electronic supplementary material (ESM) Fig. 1.

### Measurement of outcome: MCE

In the STABILITY trial, MCE included death from CHD, non-fatal myocardial infarction and urgent coronary revascularisation for myocardial ischaemia. Crucially, MCE differs from the primary endpoint because it excludes strokes. MCE in GoDARTS participants was obtained from a combination of hospital admission records (non-fatal MCE) and official deaths records using International Classification of Disease (ICD-10) codes (http://apps.who.int/classifications/icd10/browse/2016/en). All codes capturing ischaemic heart disease were used; these included myocardial infarctions and coronary artery disease, which contributed the majority of events.

### Covariates

Potential confounders considered were age, sex, smoking status, lipid levels (non-HDL-c) and hypertension. In GoDARTS, the use of hypertension-, lipid- and diabetes-controlling medications were found to be more informative predictors of MCE compared with clinical measures. Therefore, medication use was treated as proxies of dyslipidaemia, hypertension and diabetes. Information on medication use was available from community prescribing records [12]. All models are adjusted for confounders. For STABILITY, all information was captured at baseline. Since STABILITY was performed on individuals with stable CHD, variables in the SMART (Second manifestations of arterial disease) risk score were used to adjust models. These included: age, sex, smoking status, hypertension, high-sensitivity C-reactive protein (CRP), eGFR, HDL-c and total cholesterol, and history of cardiovascular events [17]. All cumulative incidence graphs represent the main (unadjusted) effects, while the adjusted effects are presented in tables and through the text.

### Statistical analysis

Individuals with missing data for covariates were minimal, assumed missing at random, and excluded on a per analysis basis. Differences in these covariates across the two studies were examined using *t* tests for continuous variables and χ^2^ tests for categorical variables. Survival analyses were undertaken in both studies using Cox proportional hazards models. Proportional hazards assumptions were tested for all adjusted models presented using the LIFETEST procedure in SAS 9.4 (SAS Institute, Cary, NC, USA). First interaction analyses using continuous Lp-PLA2 activity and diabetes status were undertaken. Subsequently, interactions were tested between quartile divided Lp-PLA2 and diabetes status (using the lowest quartile of Lp-PLA2 and well-controlled or no diabetes as the reference categories). These interactions demonstrate hazards for those with equivalent LpPLA2 activity levels across diabetes status, providing an estimate of the hazards associated with poorly controlled compared with well-controlled diabetes, or diabetes compared with no diabetes. In STABILITY these interaction analyses were performed separately for the darapladib and placebo-treated arms of the trial. Second, the modifying effect of diabetes status on Lp-PLA2 activity was further explored in models stratified by diabetes status in GoDARTS and STABILITY. Informed by these analyses, risk groups were created to determine those at increased risk of MCE on the basis of their Lp-PLA2 activity and diabetes status, and to determine who would benefit most from Lp-PLA2 inhibition. Interaction between Lp-PLA2 inhibition (darapladib vs placebo) and diabetes status was examined in those with high Lp-PLA2 activity to demonstrate the effect of Lp-PLA2 inhibition in the hypothesised risk group. All analyses were performed using SAS 9.4. Plots were produced using the ‘survminer’ package in R [18]. All *p* values are unadjusted for multiple comparisons.

## Results

### Study population

In GoDARTS, 7420 participants with a recorded diagnosis of type 2 diabetes at baseline had adequate serum for Lp-PLA2 activity measurement. Of these, 1979 individuals were classified as having well-controlled diabetes and 5441 had poorly controlled diabetes. The median follow-up in GoDARTS was 11 years. The total number of MCE in the cohort was 2230. In STABILITY, 9839 trial participants did not have diabetes at recruitment, while 5989 had type 2 diabetes (ESM Fig. 1). The median follow-up in STABILITY was 3.7 years. The total number of MCE in the trial was 1551. In GoDARTS, Lp-PLA2 activity was divided into quartiles as follows: quartile 1 (Q1) ≤ 97.2 (median Q1 = 84.4), Q2 ≤ 117.3 (median Q2 = 107.5), Q3 ≤ 140.7 (median Q3 = 128.2) and Q4 ≤ 377.9 (median Q4 = 160) nmol min^−1^ ml^−1^. In the STABILITY trial, Lp-PLA2 was divided into quartiles as follows: Q1 ≤ 141.1 (median Q1 = 122.7), Q2 ≤ 169.3 (median Q2 = 155.6), Q3 ≤ 200.2 (median Q3 = 128.2) and Q4 ≤ 300.1 (median Q4 = 225.5) nmol min^−1^ ml^−1^.

### Baseline demography

Comparisons were made within each study, across diabetes status: well-controlled compared with poorly controlled type 2 diabetes in GoDARTS; and those with and those without type 2 diabetes in STABILITY (Table 1). Mean Lp-PLA2 did not differ across the comparison groups in GoDARTS (~120 nmol min^−1^ ml^−1^), while they did differ in the STABILITY trial, with the group without diabetes having higher activity levels (175.5 ± 45.2 vs 167.0 ± 46.9 nmol min^−1^ ml^−1^). The mean age differed by diabetes regulation status in GoDARTS; those with well-controlled diabetes were older than those with poorly controlled diabetes (67 years vs 65 years). Average age in STABILITY was 64 years in both groups. The proportion of male to female participants did not differ in GoDARTS, while in STABILITY there were more female participants in the diabetes group. Higher BMI was associated with poorly controlled diabetes in GoDARTS and with type 2 diabetes in the STABILITY trial. LDL-c only differed in STABILITY, with higher levels observed in the non-diabetes group. HDL-c levels were higher in the groups with well-controlled diabetes and in those without diabetes in GoDARTS and STABILITY, respectively. Triacylglycerol levels were higher in the groups with poorly controlled diabetes and type 2 diabetes in GoDARTS and STABILITY respectively. In GoDARTS, a cohort of individuals with type 2 diabetes, only 26.7% had well-controlled diabetes; in the STABILITY trial, 38% of the participants were in the diabetes group. An even proportion of participants received the Lp-PLA2 inhibitor, darapladib, across the two groups in STABILITY; 38% of those on darapladib and 37.7% on placebo had type 2 diabetes requiring pharmacotherapy. Nearly 50% of those with and without diabetes were randomised to receive darapladib.

**Table 1:** Baseline characteristics of the GoDARTS study cohort and STABILITY trial

Study subgroups	Lp-PLA2 (nmol min^−1^ ml^−1^)	Age (years)	Sex (% women)	BMI (kg/m^2^)	LDL-c (mmol/l)	HDL-c (mmol/l)	Triacylglycerol (mmol/l)	Smoking status (% ever smokers)	Distribution (%)	Lp-PLA2 inhibitor (darapladib) %
GoDARTS										
Well-controlled diabetes (*n* = 1979)	120.5 ± 35	67 ± 11*	56.4	31 ± 6*	2.1 ± 0.8	1.4 ± 0.4*	1.9 (1.1)*	60*	26.7*	–
Poorly controlled diabetes (*n* = 5441)	121.6 ± 35	65 ± 11	56.1	32 ± 6	2.1 ± 0.8	1.3 ± 0.4	2.3 (1.3)	63	73.3	–
STABILITY										
No diabetes (*n* = 9839)	175.5 ± 45.2^†^	64 ± 10^†^	17.3^†^	28.1 ± 4.6^†^	2.3 ± 0.9^†^	1.2 ± 0.3^†^	1.7 (1.1)^†^	70.4^†^	62.2^†^	49.9
Type 2 diabetes (*n* = 5989)	167.0 ± 46.9	64 ± 9	21.1	30.3 ± 5.5	2.1 ± 0.8	1.2 ± 0.3	2.0 (1.6)	67.1	37.8	50.3

Data are presented as mean ± SD, median (interquartile range) or %

*p < 0.05 vs poorly controlled diabetes group in the GoDARTS study

^†^*p < *0.05 vs type 2 diabetes group in the STABILITY trial

### Preliminary analysis of effect of Lp-PLA2 effect on MCE

Replication of the established effect between Lp-PLA2 activity and MCE risk was undertaken using both linear and quartile divided Lp-PLA2 levels in GoDARTS. Both approaches showed significant associations with MCE. In GoDARTS and STABILITY, Lp-PLA2 activity per SD increased hazards of MCE by 1.10 and 1.30 respectively (ESM Tables 1 and 2). The highest quartile compared with the lowest quartile was associated with 1.26 (95% CI 1.10, 1.46) and 1.76 (95% CI 1.33, 2.33) higher risk of MCE in GoDARTS and STABILITY, respectively, in fully adjusted models (ESM Tables 3 and 4; ESM Fig. 2).

### Interaction between Lp-PLA2 activity and diabetes status on risk of MCE in GoDARTS and STABILITY

There was a significant interaction between linear Lp-PLA2 activity and diabetes control status in predicting MCE in GoDARTS and the placebo-treated arm of the STABILITY trial (see ESM Tables 5 and 6). Similarly, models demonstrating the interaction between quartiles of Lp-PLA2 activity and diabetes control were consistently significant in both GoDARTS and STABILITY (*p*_interaction_ < 0.005) (see ESM Tables 7 and 8). The interaction between Lp-PLA2 quartiles and diabetes status in the darapladib-treated arm was not significant (ESM Table 9). Further analyses were performed using quartiles of Lp-PLA2 activity.

### Stratification of association between Lp-PLA2 and MCE by diabetes status

The effect of successive quartiles of Lp-PLA2 activity on the risk of MCE was compared across the stratifying feature, diabetes status in the two study populations (ESM Tables 10 and 11). In GoDARTS, among those with poorly controlled diabetes, Lp-PLA2 activity in the highest quartile (Q4) compared with the lowest quartile (Q1) was associated with 1.35 higher hazards of MCE (95% CI 1.16, 1.57). By comparison, among those with well-controlled diabetes Q4 vs Q1 did not significantly increase hazards of MCE (HR 1.10 [95% CI 0.80, 1.40]; ESM Table 10 and ESM Fig. 3). In STABILITY, among participants with diabetes, activity in Q4 vs Q1 was associated with 2.50 times the hazards of MCE (95% CI 1.70, 3.68) (ESM Table 11; ESM Fig. 4). Whereas, among participants without diabetes, Q4 vs Q1 showed a more modest and non-significant increased risk of MCE (1.27 [95% CI 0.97, 1.96]).

### Creating risk groups: Lp-PLA2 Q4 vs Q1–3 and diabetes status in GoDARTS

The stratified effects demonstrate that the effect of Lp-PLA2 activity is driven by diabetes status and Lp-PLA2 activity in the highest quartile (Q4), therefore activity in the lower three quartiles (Q1–3) were combined. This helps reduce the number of comparison groups, prevents inflation of results and simplifies the analysis. In GoDARTS, the higher risk group were participants with poorly controlled diabetes and Lp-PLA2 activity in Q4, while the lower risk group were participants with well-controlled diabetes and LpPLA2 activity in Q1–Q3. The higher risk group had increased hazards of MCE compared with those with poorly controlled diabetes and Lp-PLA2 activity in Q1–3 (HR 1.19 [95% CI 1.07, 1.33; *p* < 0.001]) and HR 1.29 (95% CI 1.12, 1.47) *p* < 0.001, compared with those in the lower risk group (Fig. 1; Table 2). Analyses demonstrated that, for those with well-controlled diabetes, there was no risk associated with high Lp-PLA2 activity. Models were adjusted for sex, age, smoking status, use of lipid-, diabetes- and hypertension-controlling medication.

**Table 2:** Hazards in stratified risk groups based on Lp-PLA2 activity and diabetes status in GoDARTS (Fig. 1)

Variables	Full population (*n* = 7419) HR (95% CI)	Poorly controlled diabetes: HbA_1c_ ≥ 48 mmol/mol or ≥6.5% (*n* = 5441) HR (95% CI)	Well-controlled diabetes: HbA_1c_ < 48 mmol/mol or <6.5% (*n* = 1979) HR (95% CI)
Well-controlled diabetes + Lp-PLA2 Q1–3	Reference group	–	Reference group
Well-controlled diabetes + Lp-PLA2 Q4	1.11 (0.92, 1.35)	–	1.06 (0.85, 1.33)
Poorly controlled diabetes + Lp-PLA2 Q1–3	1.03 (0.92, 1.16)	Reference group	–
Poorly controlled diabetes + Lp-PLA2 Q4	1.29 (1.12, 1.47)**	1.19 (1.07, 1.33)**	–
Lipid-controlling medication (Yes/No)	1.48 (1.25, 1.76)**	1.66 (1.36, 2.09)**	1.24 (0.93, 1.66)
Diabetes-controlling medication (Yes/No)	1.41 (1.27, 1.57)**	1.55 (1.34, 1.80)**	1.33 (1.13, 1.57)*
Hypertension-controlling medication (Yes/No)	1.66 (1.46, 1.90)**	1.56 (1.35, 1.81)**	2.20 (1.60, 3.03)**
Age (years)	1.05 (1.04, 1.05)**	1.04 (1.04, 1.05)**	1.06 (1.05, 1.07)**
Smoking status (Yes/No)	1.30 (1.19, 1.42)**	1.36 (1.22, 1.51)**	1.29 (1.07, 1.54)*
Sex (male vs female)	1.29 (1.18, 1.40)**	1.25 (1.13, 1.38)**	1.45 (1.21, 1.72)**
Non-HDL-c (mmol/l)	0.97 (0.91, 1.03)	0.92 (0.89, 1.06)	0.90 (0.81, 1.05)

Proportional hazards assumptions met (*p* > 0.05)

*p < 0.01; **p  < 0.001

### Effect of Lp-PLA2 inhibitor (darapladib) on risk of MCE in STABILITY taking into consideration baseline Lp-PLA2 activity and diabetes status

**Fig. 2:**
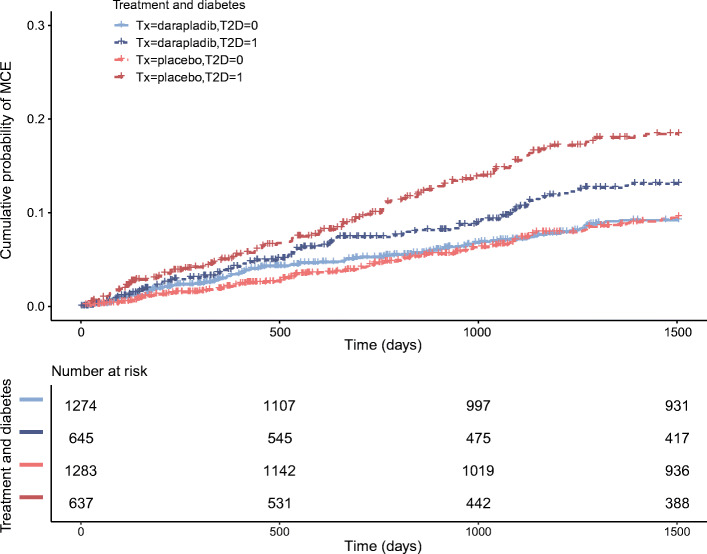
STABILITY trial: effect of Lp-PLA2 inhibitor (darapladib) therapy in the highest quartile of Lp-PLA2 activity (Q4) by diabetes status. T2D = 0 if no type 2 diabetes (T2D), T2D = 1 if type 2 diabetes; Tx, treatment. HR for Lp-PLA2 inhibition in those with type 2 diabetes 0.67 (95% CI 0.50, 0.90) *p* = 0.008. HR for Lp-PLA2 inhibition in those with no type 2 diabetes 0.96 (95% CI 0.74, 1.26) *p* = 0.78

Based on the stratified observational analysis above, we hypothesised that in the STABILITY trial among those with high Lp-PLA2 activity (Q4, *n* = 3835), participants with diabetes would benefit more from treatment with an Lp-PLA2 inhibitor than those without diabetes (Fig. 2). Interaction between Lp-PLA2 inhibition and diabetes status was tested and found to be non-significant (Wald χ^2^ = 2.85, *df* = 1, *p* = 0.09). However, Lp-PLA2 inhibition reduced risk of MCE in those with diabetes (HR 0.67 [0.50, 0.90]; *p* = 0.008) compared with those without diabetes (full model in ESM Table 12 and stratum-wise effect in Table 3).

**Table 3:** Event rate by diabetes and treatment status across quartiles

Lp-PLA2 quartiles	Placebo *n*_MCE_/*n* (%)	Darapladib *n*_MCE_/*n* (%)	Treatment effect HR (95% CI)	*p* value	*p*_interaction_^a^ Placebo-treated arm^b^	*p*_interaction_^a^ Darapladib-treated arm^c^
Type 2 diabetes						
Overall effect	286/2876 (9.9)	259/2930 (8.8)	0.89 (0.75, 1.05)	0.15^d^	–	–
Lp-PLA2 Q1	64/859 (7.5)	71/879 (8.1)	1.04 (0.74, 1.46)	0.82	0.004^b^	0.90^c^
Lp-PLA2 Q2	60/754 (8.0)	62/740 (8.4)	1.04 (0.73, 1.48)	0.84		
Lp-PLA2 Q3	60/626 (9.6)	53/666 (8.0)	0.86 (0.59, 1.25)	0.43		
Lp-PLA2 Q4	102/637 (16)	73/645 (11.3)	0.67 (0.50, 0.90)	0.008^d^		
No diabetes						
Overall effect	319/4791 (6.7)	301/4801 (6.3)	0.94 (0.80, 1.10)	0.41		
Lp-PLA2 Q1	60/1056 (5.7)	52/1058 (4.9)	0.88 (0.61, 1.28)	0.50		
Lp-PLA2 Q2	72/1172 (6.1)	72/1192 (6.0)	1.02 (0.74, 1.42)	0.90		
Lp-PLA2 Q3	79/1280 (6.2)	72/1277 (5.6)	0.87 (0.63, 1.20)	0.40		
Lp-PLA2 Q4	108/1283 (8.4)	105/1274 (8.2)	0.96 (0.74, 1.26)	0.78		
Event rate in arm/full trial population	605/7667 (7.9)	560/7731 (7.2)	0.90 (0.81, 1.01)	0.08^d^		

Models adjusted for age, sex, smoking status (ever vs never smoker), hypertension status, HDL-c, total cholesterol, CRP, eGFR and history of cerebrovascular disease

^a^Interactions have been tested between Lp-PLA2 quartiles and diabetes status in those who were placebo-treated and in those who were darapladib-treated

^b^Full adjusted model in ESM Table 8

^c^Full adjusted model in ESM Table 9

^d^Full adjusted models in ESM Table 13

Proportional hazards assumptions met (*p* > 0.05)

In the full trial population, event rate in each quartile by treatment and diabetes status are presented in Table 3, which shows 16% of those with type 2 diabetes in Q4 receiving placebo had MCE, compared with 11.3% of those receiving darapladib. The table shows that the drug treatment effect was not apparent in those without diabetes or in any of the lower quartiles of Lp-PLA2 activity regardless of diabetes status (Table 3). Models were adjusted for age, sex, hypertension status, smoking status, HDL-c, total cholesterol, CRP and eGFR. Full models for other key populations, i.e. full trial population, type 2 diabetes population, type 2 diabetes with high Lp-PLA2 activity, and those with no diabetes with lower Lp-PLA2, are presented in ESM Table 13 along with survival plots (ESM Fig. 5).

## Discussion

We provide evidence that diabetes status modifies the effect of Lp-PLA2 activity on MCE. We were able to confirm this enhanced prognostic effect among individuals with type 2 diabetes with high Lp-PLA2 activity in the placebo arm of the STABILITY trial. We also demonstrate that the same subgroup exhibited an enhanced response to Lp-PLA2 inhibitor, darapladib. While the interaction p value for darapladib treatment by diabetes status in patients with high Lp-PLA2 (≤300.1 nmol min^−1^ ml^−1^) was not significant, a clear and consistent pattern is observed within and between the two study populations where an interaction was seen between enzyme activity and diabetes status (whether defined by type 2 diabetes status or HbA_1c_ threshold). In the higher risk group, pharmacological inhibition of Lp-PLA2 activity conferred a 33% reduction in the risk of MCE. Our results identify an at-risk group most likely to benefit from the inhibition of Lp-PLA2 activity.

The heightened effect observed in a hyperglycaemic environment is consistent with the established paradigm that both type 1 and 2 diabetes are significant and independent risk factors for coronary artery disease. It is understood that hyperglycaemia induces alterations in vascular tissue that accelerate atherosclerosis [19, 20]. One of the hypothesised pathways suggests that glycation confers increased susceptibility of LDL-c to oxidative modification [9, 10], a process that would increase the inflammation associated with unstable atherosclerotic plaques. The danger with these plaques is not related to their size but primarily to their instability or tendency to rupture [3, 21]. This is also evidenced in our analyses, where poor glycaemic control is associated with a higher event rate. In STABILITY, all participants had CHD, while GoDARTS was a more general population. We observed that the effect of higher enzymatic activity in the context of type 2 diabetes was more pronounced in STABILITY than in GoDARTS, suggesting a biological and clinical risk gradient. Furthermore, the reported stratifying effect of glycaemic control also highlights a potential reason for the failure of Mendelian randomisation studies of Lp-PLA2 activity and cardiovascular outcomes based on *PLA2G7* variants [22, 23].

An in vitro study showed that inhibition of Lp-PLA2 reduced production of oxidised LDL-c by 19.8% [5]. Members of the phospholipase A2 superfamily promote the formation of oxidised LDL, which in turn is a producer of proinflammatory mediators. It could be that in the context of metabolic disease, where the production of proinflammatory mediators such as lysophosphatidylcholine and oxidised fatty acids is enhanced, an increase in circulating Lp-PLA2 is even more damaging.

A limitation of this study is the post hoc nature of the analysis in selected endpoints. However, this subgroup analysis in STABILITY was driven by the findings in GoDARTS. Another limitation is the use of different definitions of diabetes status. Well-controlled diabetes (the definition used in GoDARTS) refers to the achievement of target HbA_1c_ levels that markedly reduce risk of microvascular and macrovascular complications [24, 25]. Risk of poor outcomes for those with diabetes who achieve this degree of glycaemic control is reported to be similar to those without diabetes [26–30]. Using an HbA_1c_-based definition in STABILITY would mean limiting our analyses to just the participants with diabetes (ESM Fig. 1). The use of a surrogate and weaker definition for diabetes status likely weakens the result leading to an underestimation of the effect.

While marginal efficacy was observed in the original STABILITY analysis for MCE, this was not the case in the acute coronary syndrome trial SOLID-TIMI 52 [7]. We hypothesise this may have been because the drug was administered too late after the event (10–14 days) or because Lp-PLA2 may be less relevant in the acute setting. Crucially, STABILITY and SOLID-TIMI 52 are not comparable due to their different designs and objectives. Together with absence of effect in SOLID-TIMI 52 and the difficulties of performing a prognostic analysis in the acute setting, we chose to focus on the STABILITY trial. We focused on MCE in STABILITY as no effect on strokes (included in MACE) was observed in the trial. Observational studies have consistently reported an association between Lp-PLA2 activity and risk of CHD, while showing no evidence of greater risk of ischaemic strokes with increased Lp-PLA2 activity (RR 1.08 [95% CI 0.97, 1.20]) [1, 2, 31]. From a genetic perspective, analysis of an Lp-PLA2 null variant within the Kadoorie study also demonstrated no effect on ischaemic stroke [32].

A crucial strength of the reported findings is that the results were independent of all clinical risk factors based on the SMART risk score [17]. This implies that Lp-PLA2 activity is a significant and independent predictor of coronary events for individuals with type 2 diabetes. Together with the pharmacological effect of Lp-PLA2 inhibition, these results suggest there is value in the measurement of enzymatic activity for individuals with diabetes for risk prediction. It is worth noting that the effects persisted even though Lp-PLA2 activity was different in these two study populations, with STABILITY having higher activity.

The results presented in this manuscript offer the intriguing possibility of a precision medicine approach, in what is still a substantial patient population. The frequency of a loss-of-function variant in the gene *PLA2G7*, which results in 50% lower Lp-PLA2 activity, varies significantly by ethnicity [33]. This highlights the value in exploring the comparative risk reduction through Lp-PLA2 inhibition in different ethnicities. The development of new cardiovascular therapies over and above cholesterol-lowering drugs and platelet aggregation inhibitors has been extremely challenging in recent years. One factor here is the vast heterogeneity in patients with type 2 diabetes, which has up to this point mainly been countered with polypharmacy. In the STABILITY trial, patients were on an average of nine medications throughout the trial. It is possible that novel therapies may only be successful if the most appropriate patients can be identified. Unfortunately, in most instances the most relevant patient population can only be identified after trials have completed which, given the extended timelines of cardiovascular outcome trials, will be too late. One current example of this approach is the DalGene trial, which is utilising a genetic biomarker to stratify patients (ClinicalTrials.gov registration no. NCT02525939).

Our results show that individuals most likely to benefit from inhibition of Lp-PLA2 are those with high activity and type 2 diabetes. However, while not supported by the trial data, it is possible that in a diabetic population, those with intermediate levels of Lp-PLA2 activity may also benefit from Lp-PLA2 inhibition. However, further appropriately designed RCTs would be required to determine this. At present, it is not possible to detangle whether the potential treatment interaction is in fact specific to Lp-PLA2 as it is possible that the effect relates to the increased absolute risk in participants with type 2 diabetes. The hypothesis that darapladib would benefit patients with diabetes and high Lp-PLA2 activity needs to be tested in a new prospective RCT. A trial designed to analyse the impact of different thresholds of glycaemic control on the association between Lp-PLA2 activity, inhibition and risk of cardiovascular outcomes would be valuable. This would enable precise delivery of preventative care to those most likely to benefit from it.

## Supplementary Information

ESM (PDF 1464 kb)

## Data Availability

STABILITY trial data are available from clinicaltrials.gov repository through the GlaxoSmithKline clinical study register https://clinicaltrials.gov/ct2/show/NCT00799903. GoDARTS datasets generated during and/or analysed during the current study are available following request to the GoDARTS Access Managements Group https://godarts.org/scientific-community/.

## Abbreviations

CRP: C-reactive protein
GoDARTS: Genetics of Diabetes Audit and Research in Tayside Scotland study
HDL-c: HDL-cholesterol
LDL-c: LDL-cholesterol
Lp-PLA2: Lipoprotein-associated phospholipase A2
MACE: Major adverse cardiovascular events
MCE: Major coronary events
SOLID-TIMI 52: Stabilization of Plaque Using Darapladib-Thrombolysis in Myocardial Infarction 52
STABILITY: Stabilization of Atherosclerotic Plaque by Initiation of Darapladib Therapy

## References

[1] Packard CJ, O’Reilly DSJ, Caslake MJ (2000). Lipoprotein-associated phospholipase A_2_ as an independent predictor of coronary heart disease. N Engl J Med.

[2] The Lp-PLA2 Studies Collaboration (2010). Lipoprotein-associated phospholipase A_2_ and risk of coronary disease, stroke, and mortality: collaborative analysis of 32 prospective studies. Lancet.

[3] Wilensky RL, Shi Y, Mohler ER (2008). Inhibition of lipoprotein-associated phospholipase A2 reduces complex coronary atherosclerotic plaque development. Nat Med.

[4] Tellis C, Tselepis A (2014). Pathophysiological role and clinical significance of lipoprotein-associated phospholipase A2 (Lp-PLA2) bound to LDL and HDL. Curr Pharm Des.

[5] Jackisch L, Kumsaiyai W, Moore JD (2018). Differential expression of Lp-PLA2 in obesity and type 2 diabetes and the influence of lipids. Diabetologia.

[6] The STABILITY Investigators (2014). Darapladib for preventing ischemic events in stable coronary heart disease. N Engl J Med.

[7] O’Donoghue ML, Braunwald E, White HD (2014). Effect of darapladib on major coronary events after an acute coronary syndrome: the SOLID-TIMI 52 randomized clinical trial. JAMA.

[8] Siddiqui MK, Kennedy G, Carr F (2018). Lp-PLA2 activity is associated with increased risk of diabetic retinopathy: a longitudinal disease progression study. Diabetologia.

[9] Bowie A, Owens D, Collins P, Johnson A, Tomkin GH (1993). Glycosylated low density lipoprotein is more sensitive to oxidation: implications for the diabetic patient?. Atherosclerosis.

[10] Steinbrecher UP, Witztum JL (1984). Glucosylation of low-density lipoproteins to an extent comparable to that seen in diabetes slows their catabolism. Diabetes.

[11] He S, Chousterman BG, Fenn A (2015). Lp-PLA_2_ antagonizes left ventricular healing after myocardial infarction by impairing the appearance of reparative macrophages. Circ Heart Fail.

[12] Hébert HL, Shepherd B, Milburn K (2018). Cohort profile: genetics of diabetes audit and research in Tayside Scotland (GoDARTS). Int J Epidemiol.

[13] Wallentin L, Held C, Armstrong PW et al (2016) Lipoprotein-associated phospholipase A2 activity is a marker of risk but not a useful target for treatment in patients with stable coronary heart disease. J Am Heart Assoc 5(6):e00347. 10.1161/JAHA.116.003407 10.1161/JAHA.116.003407 PMC4937279 27329448

[14] Zhuo S, Wolfert RL, Yuan C (2017). Biochemical differences in the mass and activity tests of lipoprotein-associated phospholipase A2 explain the discordance in results between the two assay methods. Clin Biochem.

[15] Topbas C, Swick A, Razavi M, Anderson NL, Pearson TW, Bystrom C (2018). Measurement of lipoprotein-associated phospholipase A2 by use of 3 different methods: exploration of discordance between ELISA and Activity assays. Clin Chem.

[16] WHO (2019). Classification of diabetes mellitus.

[17] Dorresteijn JAN, Visseren FLJ, Wassink AMJ (2013). Development and validation of a prediction rule for recurrent vascular events based on a cohort study of patients with arterial disease: the SMART risk score. Heart.

[18] Kassambara A, Kosinski M, Biecek P (2019) ggsurvplot: Drawing Survival Curves. Available from: www.sthda.com/english/rpkgs/survminer/. Accessed: 1 July 2020

[19] Aronson D, Rayfield EJ, Stamler J (1993). Role of cardiovascular risk factors in prevention and treatment of macrovascular disease in diabetes. Diabetes Care.

[20] Tanaka A, Miyauchi K, Mokuno H, Daida H (2005). Pathogenesis of atherosclerosis in diabetes mellitus. Respiration and Circulation.

[21] Webb NR (2008). Getting to the core of atherosclerosis. Nat Med.

[22] Grallert H, Dupuis J, Bis JC (2012). Eight genetic loci associated with variation in lipoprotein-associated phospholipase A2 mass and activity and coronary heart disease: meta-analysis of genome-wide association studies from five community-based studies. Eur Heart J.

[23] Chu AY, Guilianini F, Grallert H (2012). Genome-wide association study evaluating lipoprotein-associated phospholipase A2 mass and activity at baseline and after rosuvastatin therapy. Circ Cardiovasc Genet.

[24] Cosentino F, Grant PJ, Aboyans V et al (2019) 2019 ESC Guidelines on diabetes, pre-diabetes, and cardiovascular diseases developed in collaboration with the EASD. Eur Heart J 255–323. 10.1093/eurheartj/ehz486 10.1093/eurheartj/ehz486 31497854

[25] International Diabetes Federation (2017) IDF Clinical Practice Recommendations for managing Type 2 Diabetes in Primary Care International Diabetes Federation. Available from: www.idf.org/e-library/guidelines/128-idf-clinical-practice-recommendations-for-managing-type-2-diabetes-in-primary-care.html. Accessed: 1 June 2019

[26] American Diabetes Association (2004). Screening for type 2 diabetes. Diabetes Care.

[27] Lebovitz HE, Austin MM, Blonde L (2006). ACE/AACE consensus conference on the implementation of outpatient management of diabetes mellitus: consensus conference recommendations. Endocr Pract.

[28] Monnier L, Colette C (2009). Target for glycemic control: concentrating on glucose. Diabetes Care.

[29] Ceriello A, Colagiuri S (2008). International diabetes federation guideline for management of postmeal glucose: a review of recommendations. Diabet Med.

[30] NHS (2018) Hyperglycaemia (high blood sugar). Available from: www.nhs.uk/conditions/high-blood-sugar-hyperglycaemia/. Accessed: 1 June 2019

[31] Hu G, Liu D, Tong H, Huang W, Hu Y, Huang Y (2019). Lipoprotein-associated phospholipase A2 activity and mass as independent risk factor of stroke: a Meta-analysis. Biomed Res Int.

[32] Millwood IY, Bennett DA, Walters RG (2016). A phenome-wide association study of a lipoprotein-associated phospholipase a 2 loss-of-function variant in 90 000 Chinese adults. Int J Epidemiol.

[33] Wang Q, Hao Y, Mo X (2010). PLA2G7 gene polymorphisms and coronary heart disease risk: a meta-analysis. Thromb Res.

