# Supplementary material for: Diabetes status modifies the long-term effect of lipoprotein-associated phospholipase A2 on major coronary events
Source: Diabetologia. 2021 Sep 25;65(1):101–12. doi: 10.1007/s00125-021-05574-5 (PMC8660745; doi:10.1007/s00125-021-05574-5)
Supplement: Supplementary file 1 — (PDF 1464 kb) [file 125_2021_5574_MOESM1_ESM.pdf]

Online-only supplemental materials:

Diabetes status modifies the long-term effect of Lp-PLA_2 on major coronary events

M K Siddiqui et al.

Contents

Tables

ESM Table S1. Main effect of LpPLA2 per standard deviation on MCE in the GoDARTS cohort (n=6,159)
ESM Table 2. Main effect of LpPLA2 per standard deviation on MCE in placebo treated STABILITY participants (n=7,109)
ESM Table 3. Main effect of LpPLA2 (in quartiles) on MCE in the GoDARTS cohort (n= 6,159)
ESM Table 4. Effect of LpPLA2 (in quartiles) on MCE in placebo treated STABILITY trial participants (n= 7109)
ESM Table 5. Interaction effect between linear Lp-PLA2 activity and diabetes-control status on the occurrence of MCE in the GoDARTS cohort (n = 7,419*)
ESM Table 6. Interaction effect between linear Lp-PLA2 activity and diabetes status) on the occurrence of MCE in the STABILITY trial (n = 7,109)
ESM Table 7. Interaction effect between quartiles of Lp-PLA2 activity and diabetes status) on the occurrence of MCE in the GoDARTS cohort (n = 7,419).
ESM Table 8. Interaction effect between quartiles of Lp-PLA2 activity and diabetes status on the occurrence of MCE in the placebo-treated arm of the STABILITY trial (n = 7,109).
ESM Table 9. Interaction effect between quartiles of Lp-PLA2 activity and diabetes status on the occurrence of MCE in the darapladib-treated arm of the STABILITY trial (n=7,157)
ESM Table 10. Stratified effect of successive quartiles of Lp-PLA2 activity on MCE by diabetes-control status in GoDARTS
ESM Table 11. Stratified effect of successive quartiles of Lp-PLA2 activity on MCE by type 2 diabetes status in prognostic arm of STABILITY trial
ESM Table 12: Effect of LpPLA2 inhibition in STABILITY participants with high Lp-PLA2 activity (Q4)– main text Figure 2
ESM Table 13. Association table for drug effect (Lp-PLA2 inhibition) in STABILITY sub-groups – main text Figure 3

Figures

ESM Fig 1. Population flow chart describing both study populations, GoDARTS upper panel and STABILITY lower panel.
ESM Fig 2. Demonstrating association between population-specific, successive Lp-PLA2 quartiles and MCE in GoDARTS (2a) and placebo-treated participants in STABILITY (2b)
ESM Fig 3. Stratified effect of successive Lp-PLA2 quartiles on the risk of MCE by diabetes control status in GoDARTS
ESM Fig 4. STABILITY trial: Hazards of increasing quartiles of Lp-PLA2 activity on the risk of MCE in participants with type 2 diabetes receiving placebo

ESM Table S1. Main effect of LpPLA2 per standard deviation on MCE in the GoDARTS cohort (n=6,159)

	**Hazard Ratio (95%CI)**	**P value**
LpPLA2 (per SD)	1.09 (1.03, 1.15)	0.002
Lipid-controlling medication (Yes/No)	1.50 (1.25, 1.79)	<0.0001
Diabetes-controlling medication (Yes/No)	1.46 (1.30, 1.63)	<0.0001
Hypertension controlling medication (Yes/No)	1.68 (1.45, 1.95)	<0.0001
Age (years)	1.05 (1.048, 1.054)	<0.0001
Smoking status (Yes/No)	1.28 (1.16, 1.41)	<0.0001
Sex (F v M)	1.30 (1.18, 1.44)	<0.0001
Non-HDL cholesterol (mmol/L)	0.96 (0.89, 1.03)	0.32

Proportional hazards assumptions met (P value > 0.05).

ESM Table 2. Main effect of LpPLA2 per standard deviation on MCE in placebo treated STABILITY participants (n=7,109)

	**Hazard Ratio (95%CI)**	**P value**
LpPLA2 (per SD)	1.30 (1.17, 1.44)	<0.0001
Age (years)	1.01 (1.002, 1.023)	0.02
Sex (F v M)	0.91 (0.72, 1.16)	0.45
Smoking status (Yes v.No)	1.40 (1.15, 1.71)	0.0009
Hypertension (Yes v No)	1.30 (1.03, 1.63)	0.02
HDL cholesterol (mmol/L)	1.08 (0.80, 1.46)	0.64
Cholesterol (mmol/L)	1.04 (0.94, 1.15)	0.47
High-sensitivity C-reactive Protein (mg/L)	1.013 (1.006, 1.020)	0.0005
eGFR (mL/min)	0.54 (0.39, 0.73)	<0.0001
History of Coronary Heart Disease (Yes/No)	1.12 (0.60, 2.09)	0.73
History of Cerebrovascular Disease	1.29 (0.99, 1.67)	0.06
History of Abdominal aortic aneurysm	0.61 (029, 1.29)	0.19
History of Peripheral artery disease	1.22 (0.39, 3.82)	0.73
Type 2 diabetes	1.56 (1.31, 1.85)	<0.0001

Proportional hazards assumptions met (P value > 0.05).

ESM Table 3. Main effect of LpPLA2 (in quartiles) on MCE in the GoDARTS cohort (n= 6,159)

**LpPLA2 quartiles**	**Hazard ratios (95% CI)**	**P value**
Quartile 2 v. 1	1.11 (0.97, 1.27)	0.12
Quartile 3 v. 1	1.17 (1.02, 1.35)	0.002
Quartile 4 v. 1	1.26 (1.10, 1.46)	<0.0001
Lipid-controlling medication (Yes/No)	1.49 (1.25, 1.79)	<0.0001
Diabetes-controlling medication (Yes/No)	1.46 (1.30, 1.62)	<0.0001
Hypertension controlling medication (Yes/No)	1.68 (1.45, 1.94)	<0.0001
Age (years)	1.05 (1.048, 1.053)	<0.0001
Sex (M v. F)	1.10 (0.99, 1.22)	0.05
Smoking status (Yes v.No)	1.28 (1.15, 1.41)	<0.0001
Non-HDL cholesterol (mmol/L)	0.97 (0.90, 1.03)	0.32

Proportional hazards assumptions met (P value >0.05). Model: MCE ~ Lp_PLA2 + Diabetes controlling medication + hypertension controlling medication + lipid controlling medication + smoking status + LDL-cholesterol

ESM Table 4. Effect of LpPLA2 (in quartiles) on MCE in placebo treated STABILITY trial participants (n= 7,109)

LpPLA2 quartiles	**Hazard ratios (95% CI)**	**P value**
Quartile 2 v. 1	1.10 (0.85, 1.44)	0.46
Quartile 3 v. 1	1.19 (0.91, 1.56)	0.20
Quartile 4 v. 1	1.76 (1.33, 2.33)	<0.0001
Age (years)	1.013 (1.003, 1.024)	0.014
Sex (F v M)	0.88 (0.70, 1.12)	0.31
Smoking status (Yes v.No)	1.41 (1.15, 1.72)	0.0008
Hypertension (Yes v No)	1.30 (1.04, 1.64)	0.02
HDL cholesterol (mmol/L)	1.00 (0.74, 1.36)	0.98
Cholesterol (mmol/L)	1.08 (0.98, 1.18)	0.13
High-sensitivity C-reactive Protein (mg/L)	1.013 (1.006, 1.020)	0.0004
eGFR (mL/min)	0.53 (0.39, 0.72)	<0.0001
History of Coronary Heart Disease (Yes/No)	1.13 (0.61, 2.12)	0.70
History of Cerebrovascular Disease (Yes/No)	1.30 (1.01, 1.69)	0.05
History of Abdominal Aortic Aneurysm (Yes/No)	0.62 (0.29, 1.30)	0.21
History of Peripheral artery disease (Yes/No)	1.22 (0.39, 3.81)	0.74
Type 2 diabetes (Yes/No)	1.54 (1.30, 1.83)	<0.0001

Proportional hazards assumptions met (P value =0.8)

2. Interaction between diabetes control status (in GoDARTS) and diabetes status (in STABILITY) with Lp-PLA2 activity on the risk of MCE

ESM Table 5. Interaction effect between linear Lp-PLA2 activity and diabetes-control status (HbA1c< 48 mmol/mol v. ≥ 48 mmol/mol) on the occurrence of MCE in the GoDARTS cohort (n = 7,419)

	**HR (95% CI)**	**P value**
Interaction: LpPLA2 (nmol/min/ml)*Diabetes control status	1.001 (1.0005, 1.0015)	0.004
LpPLA2 (nmol/min/ml)	1.002 (1.00, 1.002)	0.008
Diabetes control status (Poor v. well-controlled)	1.29 (1.06, 1.57)	0.013
Lipid-controlling medication (Yes/No)	1.52 (1.28, 1.80)	<0.0001
Diabetes-controlling medication (Yes/No)	1.48 (1.33, 1.64)	<0.0001
Hypertension controlling medication (Yes/No)	1.69 (1.48, 1.93)	<0.0001
Age (years)	1.05 (1.04, 1.05)	<0.0001
Smoking status (Yes/No)	1.30 (1.18, 1.42)	<0.0001
Sex (M v. F)	1.28 (1.17, 1.39)	<0.0001
Non-HDL cholesterol (mmol/L)	0.97 (0.90, 1.03)	0.32

Proportional hazards assumptions met (P value>0.05)

ESM Table 6. Interaction effect between linear Lp-PLA2 activity and diabetes status) on the occurrence of MCE in the STABILITY trial (n = 7,109)

	**HR (95% CI)**	**P value**
Interaction: LpPLA2 (nmol/min/ml)*Diabetes control status	1.004 (1.001, 1.008)	0.015
LpPLA2 (nmol/min/ml)	0.99 (0.99, 1.005)	0.77
Diabetes status (Type 2 diabetes v. no diabetes)	0.70 (0.0.36, 1.36)	0.29
Sex (M v. F)	0.91 (0.71, 1.15)	0.42
Age (years)	1.02 (1.01, 1.03)	0.02
Smoking status (Yes v.No)	1.40 (1.14, 1.70)	0.0011
Hypertension (Yes v No)	1.30 (1.04, 1.64)	0.02
HDL cholesterol (mmol/L)	1.07 (0.79, 1.44)	0.68
Cholesterol (mmol/L)	1.04 (0.94, 1.15)	0.46
High-sensitivity C-reactive Protein (mg/L)	1.013 (1.006, 1.02)	0.0004
eGFR (mL/min)	0.53 (0.39, 0.73)	<0.0001
History of Coronary Heart Disease (Yes/No)	1.134 (0.61, 2.12)	0.70
History of Cerebrovascular Disease (Yes/No)	1.29 (0.99, 1.67)	0.06
History of Abdominal Aortic Aneurysm (Yes/No)	0.61 (0.30, 1.29)	0.19
History of Peripheral artery disease (Yes/No)	1.22 (0.39, 3.38)	0.73

Proportional hazards assumptions met (P value >0.05).

ESM Table 7. Interaction effect between quartiles of Lp-PLA2 activity and diabetes status) on the occurrence of MCE in the GoDARTS cohort (n = 7,419).

Overall interaction variable type 3 test (Interaction between diabetes status in each quartile)
Wald Chi-square for interaction (Wald chi-square 15.8, DF=3, P value 0.001)

	**HR (95% CI)**	**P value**
Interaction:<br>LpPLA2 Q4+PC-T2D v.<br>LpPLA2 Q4+WC-T2D	1.20 (1.16, 1.54)	0.04
LpPLA2 Q3+ PC-T2D v.<br>LpPLA2 Q3+ WC-T2D	1.16 (0.95, 1.43)	0.14
LpPLA2 Q2+ PC-T2D v.<br>LpPLA2 Q2+ WC-T2D	1.18 (1.03, 1.36)	0.02
LpPLA2 Q1+ PC-T2D v.<br>LpPLA2 Q1+ WC-T2D	0.97 (0.79, 1.19)	0.11
LpPLA2 quartiles (Q4 v Q1)	1.12 (0.95, 1.36)	0.08
LpPLA2 quartiles (Q3 v Q1)	1.10 (0.89, 1.18)	0.12
LpPLA2 quartiles (Q2 v Q1)	1.00 (0.78, 1.13)	0.65
Diabetes status	1.04 (0.95, 1.14)	0.43
Lipid-controlling medication (Yes/No)	1.51 (1.28, 1.79)	<0.0001
Diabetes-controlling medication (Yes/No)	1.43 (1.29, 1.59)	<0.0001
Hypertension controlling medication (Yes/No)	1.70 (1.49, 1.94)	<0.0001
Age (years)	1.05 (1.04, 1.05)	<0.0001
Smoking status (Yes/No)	1.30 (1.19, 1.42)	<0.0001
Sex (M v. F)	1.28 (1.17, 1.40)	<0.0001

Proportional hazards assumptions met (P value>0.05). PC-T2D: poorly controlled diabetes, WC-T2D: well-controlled diabetes.

ESM Table 8. Interaction effect between quartiles of Lp-PLA2 activity and diabetes status on the occurrence of MCE in the placebo-treated arm of the STABILITY trial (n = 7,109).
Overall interaction variable type 3 test (Wald chi-square=8.46, DF=3, P =0.0036)

	**HR (95% CI)**	**P value**
LpPLA2 Q4+T2D v. LpPLA2 Q4+non-T2D	2.09 (1.57, 2.78)	0.0001
LpPLA2 Q3+T2D v. LpPLA2 Q3+non-T2D	1.44 (0.998, 2.06)	0.05
LpPLA2 Q2+T2D v. LpPLA2 Q2+non-T2D	1.31 (0.90, 1.89)	0.2
LpPLA2 Q1+T2D v. LpPLA2 Q1+non-T2D	1.11 (0.75, 1.65)	0.60
LpPLA2 quartiles (Q4 v Q1)	1.52 (1.21, 1.98)	0.002
LpPLA2 quartiles (Q3 v Q1)	1.17 (0.78, 1.33)	0.35
LpPLA2 quartiles (Q2 v Q1)	0.98 (0.75, 1.21)	0.95
Diabetes status	0.84 (0.53, 1.32)	0.45
Age (years)	1.02 (1.01, 1.03)	<0.0001
Sex (F v. M)	0.90 (0.71, 1.14)	0.40
Smoking status (Yes v.No)	1.40 (1.14, 1.71)	0.001
Hypertension (Yes v No)	1.31 (1.05, 1.65)	0.02
HDL cholesterol (mmol/L)	1.01 (0.75, 1.36)	0.93
Cholesterol (mmol/L)	1.07 (0.98, 1.16)	0.15
High-sensitivity C-reactive Protein (mg/L)	1.013 (1.006, 1.02)	0.0003
eGFR (mL/min)	0.53 (0.39, 0.73)	<0.0001
History of Coronary Heart Disease (Yes/No)	1.15 (0.61, 2.15)	0.67
History of Cerebrovascular Disease (Yes/No)	1.30 (0.99, 1.68)	0.05
History of Abdominal Aortic Aneurysm (Yes/No)	0.62 (0.30, 1.31)	0.21
History of Peripheral artery disease (Yes/No)	1.16 (0.37, 3.63)	0.80

Proportional hazards assumptions met (P value>0.05). Non-T2D, no diabetes.

ESM Table 9. Interaction effect between quartiles of Lp-PLA2 activity and diabetes status on the occurrence of MCE in the darapladib-treated arm of the STABILITY trial (n=7,157)
Overall interaction variable type 3 test (Wald chi-square=0.4, DF=3, P =0.8)

	**HR (95% CI)**	**P value**
LpPLA2 Q4+T2D v. LpPLA2 Q4+non-T2D	1.40 (1.02, 1.91)	0.036
LpPLA2 Q3+T2D v. LpPLA2 Q3+non-T2D	1.47 (1.01, 2.13)	0.04
LpPLA2 Q2+T2D v. LpPLA2 Q2+non-T2D	1.28 (0.88, 1.86)	0.20
LpPLA2 Q1+T2D v. LpPLA2 Q1+non-T2D	1.77 (1.19, 2.63)	0.005
LpPLA2 quartiles (Q4 v Q1)	1.22 (0.82, 1.25)	0.58
LpPLA2 quartiles (Q3 v Q1)	1.19 (0.90, 1.30)	0.08
LpPLA2 quartiles (Q2 v Q1)	1.12 (0.87. 1.44)	0.40
Diabetes status	1.48 (0.93, 2.33)	0.09
Age (years)	1.01 (1.003, 1.03)	0.02
Smoking status (Yes v.No)	1.10 (0.90, 1.34)	0.81
Hypertension (Yes v No)	1.03 (0.82, 1.28)	0.83
HDL cholesterol (mmol/L)	0.72 (0.52, 0.99)	0.04
Cholesterol (mmol/L)	1.08 (0.98, 1.18)	0.12
High-sensitivity C-reactive Protein (mg/L)	1.012 (1.003, 1.02)	0.006
eGFR (mL/min)	0.66 (0.48, 0.91)	0.01
History of Coronary Heart Disease (Yes/No)	1.66 (0.78, 3.49)	0.19
History of Cerebrovascular Disease (Yes/No)	1.51 (1.16, 1.96)	0.002
History of Abdominal Aortic Aneurysm (Yes/No)	1.35 (0.81, 2.23)	0.25
History of Peripheral artery disease (Yes/No)	3.97 (1.3, 9.66)	0.002

Proportional hazards assumptions met (P value>0.05). Sex, smoking status, hypertension, and total cholesterol were non-informative. T2D: type 2 diabetes, non-T2D: no diabetes.

3. Effect stratified by diabetes-control status (GoDARTS) or diabetes status (STABILITY)

ESM Table 10. Stratified effect of successive quartiles of Lp-PLA2 activity on MCE by diabetes-control status in GoDARTS

	**Poorly controlled diabetes. HbA1c ≥ 48 mmol/mol or ≥ 6.5% (n=5,441)**		**Well-controlled diabetes HbA1c < 48 mmol/mol or <6.5% (n=1,979)**	
**Lp-PLA$_2$ Quartiles**	**Hazard ratios (95% CI)**	**P value**	**Hazard ratios (95% CI)**	**P value**
Quartile 2 v. 1 *	1.17 (1.01, 1.36)	0.014	0.93 (0.75, 1.14)	0.19
Quartile 3 v. 1 *	1.21 (1.05, 1.41)	0.02	1.08 (0.84, 1.39)	0.54
**Quartile 4 v. 1 ***	**1.35 (1.16, 1.57)**	**<0.0001**	1.10 (0.80, 1.40)	0.68
Quartile 4 v. 2 *	1.14 (0.99, 1.33)	0.058	1.19 (0.91, 1.55)	0.98
Quartile 4 v. 3 *	1.13 (0.99, 1.29)	0.065	0.98 (0.76, 1.26)	0.21
**Quartile 4 v. 3-1 ¤**	**1.19 (1.07, 1.33)**	**0.001**	1.06 (0.85, 1.33)	0.58
Lipid-controlling medication (Yes/No)	1.66 (1.32, 2.09)	<0.0001	1.23 (0.92, 1.64)	0.16
Diabetes-controlling medication (Yes/No)	1.55 (1.34, 1.80)	<0.0001	1.33 (1.13, 1.57)	0.001
Hypertension controlling medication (Yes/No)	1.60 (1.38, 1.85)	<0.0001	2.19 (1.59, 3.02)	<0.0001
Age (years)	1.04 (1.037,1.05)	<0.0001	1.06 (1.05, 1.07)	<0.0001
Smoking status (Yes/No)	1.30 (1.17, 1.45)	<0.0001	1.29 (1.08, 1.55)	0.006
Sex (M v. F)	1.22 (1.10, 1.35)	<0.0001	1.44 (1.20, 1.72)	<0.0001
Non-HDL cholesterol (mmol/L)	0.92 (0.90, 1.06)	0.63	0.92 (0.81, 1.05)	0.83

Proportional hazards assumptions met for both groups: poor glycaemic control group P value=0.8 and good glycaemic control P value = 0.1.
*Comparisons labelled with * and ¤ were run in separate models. All other covariates are from model labelled *. P value for multiple testing of quartile effects = 0.008. Quartile comparisons in bold are significant using the threshold for multiple testing. Effect is presented graphically in ESM Figure 3.*

ESM Table 11. Stratified effect of successive quartiles of Lp-PLA2 activity on MCE by type 2 diabetes status in placebo-arm of the STABILITY trial

	**Type 2 Diabetes (n=2,872) with CRP (n=2655)**		**No Diabetes (n=4,782) with CRP (n=4,454)**	
**Lp-PLA$_2$ Quartiles**	**Hazard Ratio (95% CI)**	**P value**	**Hazard Ratio (95% CI)**	**P value**
Quartile 2 v. 1 *	1.13 (0.76, 1.66)	0.55	1.05 (0.74, 1.50)	0.70
Quartile 3 v. 1 *	1.33 (0.90, 1.97)	0.16	1.04 (0.74, 1.47)	0.81
**Quartile 4 v. 1 ***	**2.50 (1.70, 3.68)**	**<0.0001**	1.27 (0.97, 1.96)	0.09
**Quartile 4 v. 2 ***	**1.99 (1.35, 2.93)**	**0.0005**	1.16 (0.80. 1.68)	0.44
**Quartile 4 v. 3 ***	**1.77 (1.25, 2.51)**	**0.001**	1.14 (0.83, 1.58)	0.42
**Quartile 4 v. 3-1 ¤**	**2.18 (1.62, 2.93)**	**<0.0001**	1.22 (0.92, 1.62)	0.17
Age (years)	1.02 (1.001, 1.032)	0.04	1.01 (0.99, 1.03)	0.19
Sex (F v M)	0.70 (0.50, 1.01)	0.05	1.10 (0.81, 1.49)	0.55
Smoking status (Yes v.No)	1.18 (0.90, 1.55)	0.23	1.59 (1.21, 2.09)	0.0009
Hypertension (Yes v No)	1.24 (0.94,1.83)	0.28	1.40 (1.07, 1.85)	0.02
Total cholesterol (mmol/L)	1.03 (0.90, 1.17)	0.70	1.11 (0.99, 1.25)	0.08
eGFR (mL/min/1.73m^2)	0.44 (0.29, 0.68)	0.0002	0.65 (0.42, 1.04)	0.07
High-sensitivity C-reactive protein	1.013 (1.002, 1.024)	0.03	1.01 (1.005, 1.024)	0.003
History of Coronary Heart Disease (Yes/No)	1.49 (0.55, 4.002)	0.43	0.92 (0.41, 2.07)	0.83
History of Cerebrovascular Disease (Yes/No)	1.68 (1.20, 2.33)	0.002	0.92 (0.59, 1.42)	0.83
History of Abdominal Aortic Aneurysm (Yes/No)	0.54 (0.17, 1.71)	0.30	0.69 (0.26, 1.87)	0.69
History of Peripheral artery disease (Yes/No)	0.000 (0.000, 0.000)	0.96	4.45 (1.40, 14.12)	0.01

*Proportional hazards assumptions met for both groups (P value>0.5). Comparisons labelled with * and ¤ were run in separate models. All other covariates from model labelled *.*
P value for multiple testing of quartile effects = 0.008. Quartile comparisons in bold are significant using the threshold for multiple testing. Effects in T2D groups are presented graphically in ESM Figure 4.

4. Risk groups analysis -drug effect in STABILITY

ESM Table 12: Effect of LpPLA2 inhibition in STABILITY participants with high Lp-PLA2 activity (Q4) n = 3835 – main text Figure 2. Wald chi-square for interaction term=2.85, DF=1, P value =0.09

Variables	HR (95% confidence limits)	P value
Type 2 diabetes (darapladib v. placebo)	0.67 (0.50,0.90)	0.008
No diabetes (darapladib v. placebo)	0.96 (0.74,1.26)	0.78
Type 2 diabetes	2.02 (1.52, 2.69)	<0.0001
Darapladib v. placebo	0.98 (0.74, 1.30)	0.91
Age (years)	1.02 (1.00, 1.03)	0.02
Sex (F v. M)	0.97 (0.64, 1.47)	0.88
Smoking status (Yes v. No)	1.16 (0.84, 1.61)	0.36
Hypertension (Yes v. No)	1.42 (0.97, 2.08)	0.08
Total cholesterol (mmol/L)	1.102 (0.98, 1.24)	0.11
eGFR (mL/min/1.73m^2)	0.85 (0.51, 1.40)	0.52
High-sensitivity C-reactive protein	1.01 (0.996, 1.024)	0.17
History of Coronary Heart Disease (Yes/No)	1.88 (0.46, 7.60)	0.377
History of Cerebrovascular Disease (Yes/No)	1.18 (0.75, 1.85)	0.48
History of Abdominal Aortic Aneurysm (Yes/No)	0.74 (0.28, 2.01)	0.56
History of Peripheral artery disease (Yes/No)	1.03 (0.14 7.47)	0.98

Proportional hazards assumptions met (P value > 0.05).

ESM Table 13. Association table for drug effect (Lp-PLA2 inhibition) in STABILITY sub-groups – main text Figure 3

STABILITY sub-groups	n	Lp-PLA2 inhibitor (darapladib v. placebo) HR (95%CI), P value	Age (years) HR (95%CI), P value	Sex (female v. male) HR (95%CI), P value	Hyperten -sion status HR (95%CI), P value	HDL-c (mmol/L) HR (95%CI), P value	Total cholestero l HR (95%CI), P value	Smoking status HR (95%CI), P value	eGFR (mL/min /1.73m^2) HR, (95%CI), P value	History of CeVD HR, (95%CI) P value
**Figure 3a. Full population**	15,828	0.90 (0.81,1.01), 0.08	1.01 (1.01,1.03), <0.0001	0.89 (0.78,1.05), 0.17	1.25 (1.08,1.46), 0.003	0.66 (0.54,0.80), <0.0001	1.16 (1.11,1.22), <0.0001	1.27 (1.12,1.47), 0.0004	0.55 (0.44,0.68), <0.0001	1.42 (1.19,1.69), <0.0001
**Figure 3b. Type 2 diabetes**	5,989	0.89 (0.75,1.05), 0.15	1.01 (1.00,1.03), 0.013	0.80 (0.64,1.01), 0.06	1.25 (0.96,1.63), 0.09	0.69 (0.51,0.94), 0.02	1.18 (1.10,1.26), <0.0001	1.21 (1.00,1.47), 0.05	0.50 (0.38,0.67), <0.0001	1.59 (1.26,2.01), <0.0001
**Figure 3c. Higher risk group (type 2 diabetes + Lp-PLA2 in Q4)**	1,449	0.64 (0.48,0.86), 0.003*	1.02 (1.00,1.03), 0.11	0.87 (0.55,1.37), 0.54	1.59 (0.98,2.56), 0.06	0.56 (0.31,1.01), 0.05	1.15 (1.03,1.29), 0.02	1.40 (0.97,2.01), 0.07	0.75 (0.46,1.23), 0.25	1.40 (0.92,2.13), 0.12
**Figure 3d. Lower risk group (no diabetes + Lp-PLA2 in Q1-3)**	7,199	0.91 (0.75,1.11), 0.34	1.02 (1.01,1.04), <0.0001	0.98 (0.75, 1.27), 0.85	1.23 (0.97,1.56), 0.08	1.002 (0.73,1.38), 0.99	1.001 (0.88,1.14), 0.98	1.41 (1.12,1.77), 0.003	0.58 (0.40,0.86), 0.006	1.27 (0.91,1.78), 0.16

Models adjusted for age, sex, smoking status (ever v. never smoker), hypertension status, HDL-cholesterol, total cholesterol, estimated glomerular filtration rate (eGFR), and a history of cerebrovascular disease (CeVD).
**In models adjusted for C-reactive protein, the HR for ESM Fig5c (Higher Risk Group) was HR: 0.67 (0.50,0.90) P =0.008 (n=1363).*

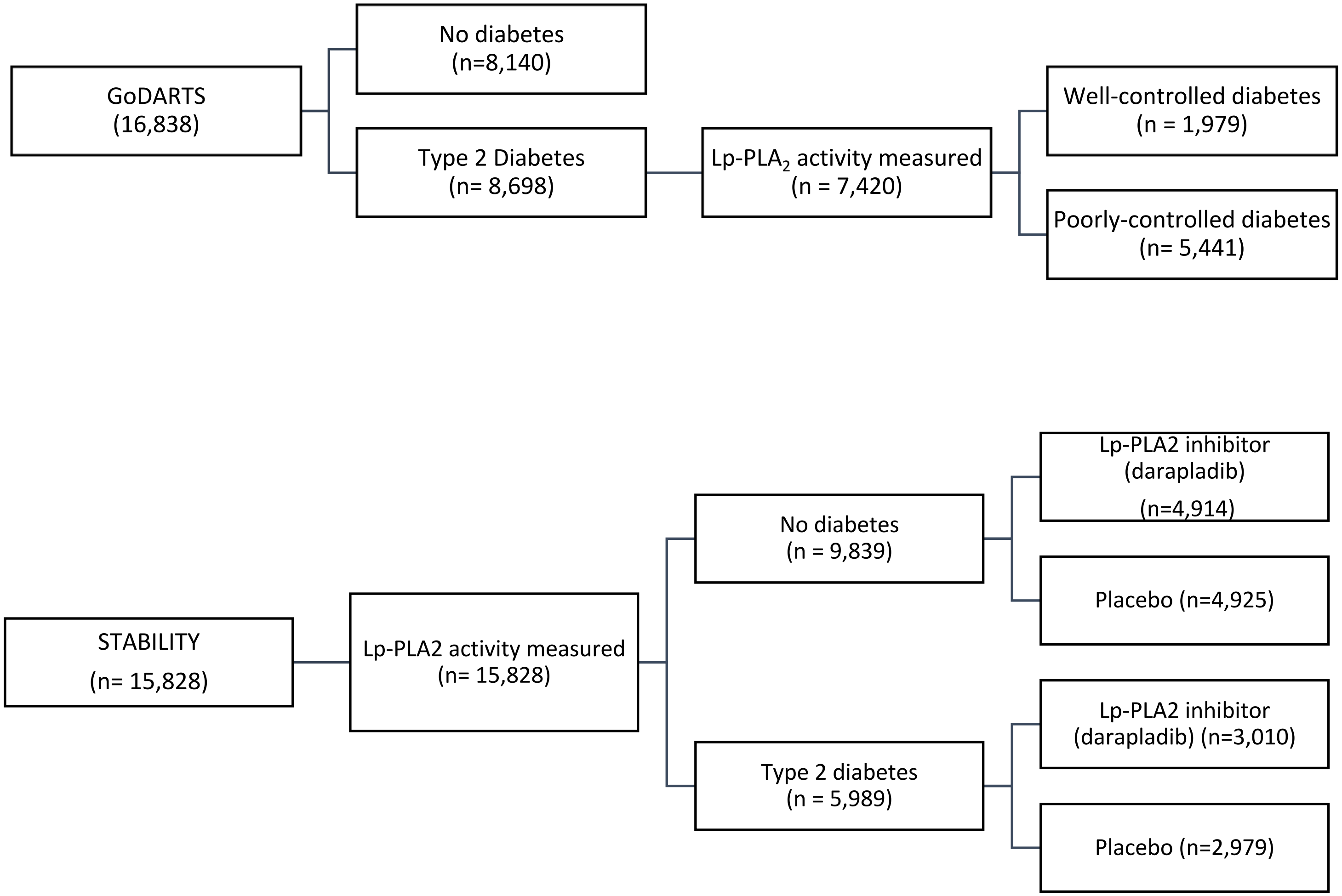

ESM Fig 1. Population flow chart describing both study populations, GoDARTS upper panel and STABILITY lower panel.

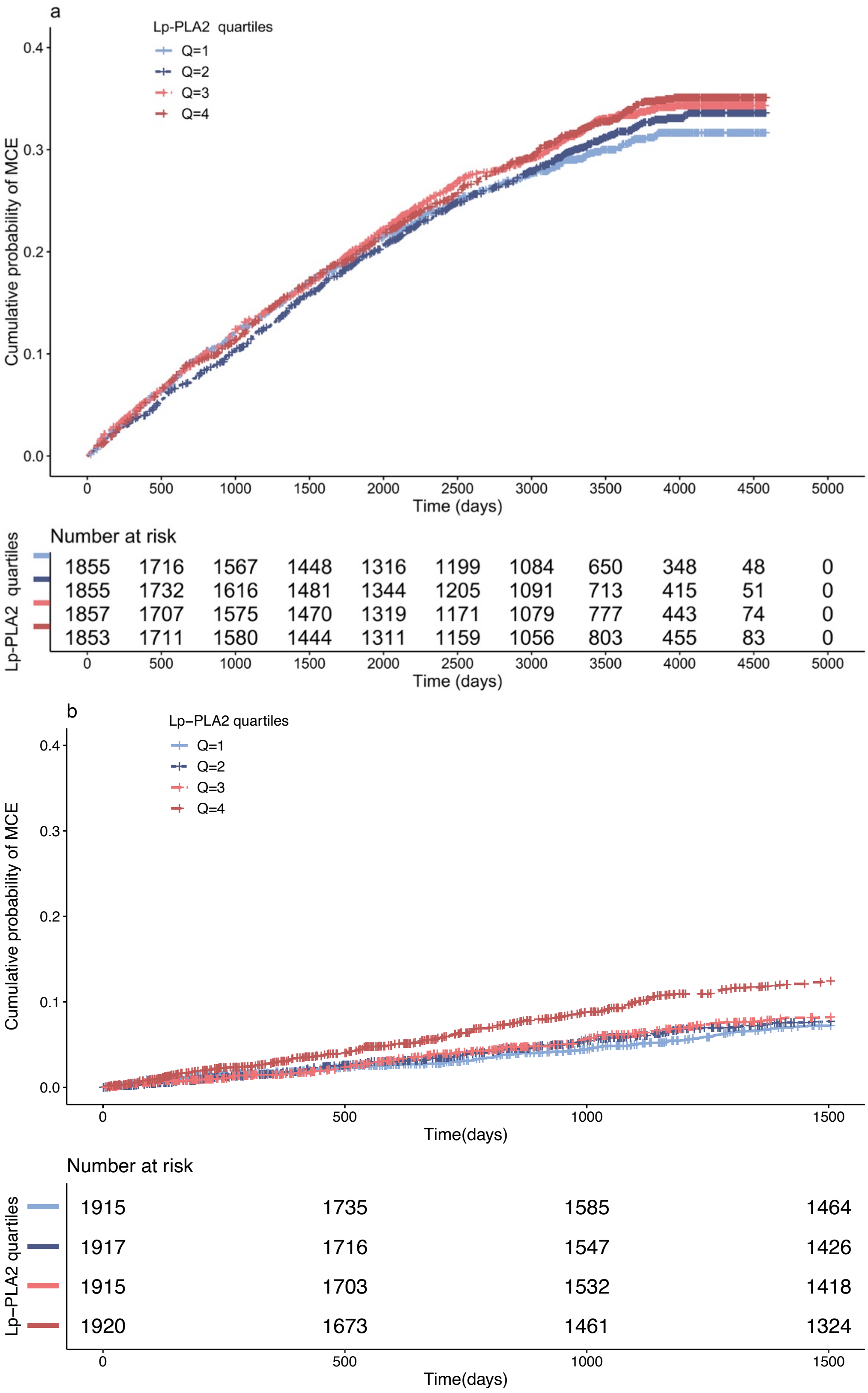

ESM Fig 2. Demonstrating association between population-specific, successive Lp-PLA2 quartiles and MCE in GoDARTS (2a) and placebo-treated participants in STABILITY (2b). 2a) Hazard ratio (95% confidence interval) for Q4 v. Q1 in GoDARTS = 1.26 (1.10, 1.46), P value <0.0001. Findings from full adjusted for GoDARTS in ESM Table 3. 2b) Hazard ratio for STABILITY Q4 v. Q1 = 1.76, (95%CI:1.33, 2.33), P< 0.0001. Findings from full adjusted model for STABILITY in ESM Table 4. These plots represent main effects.

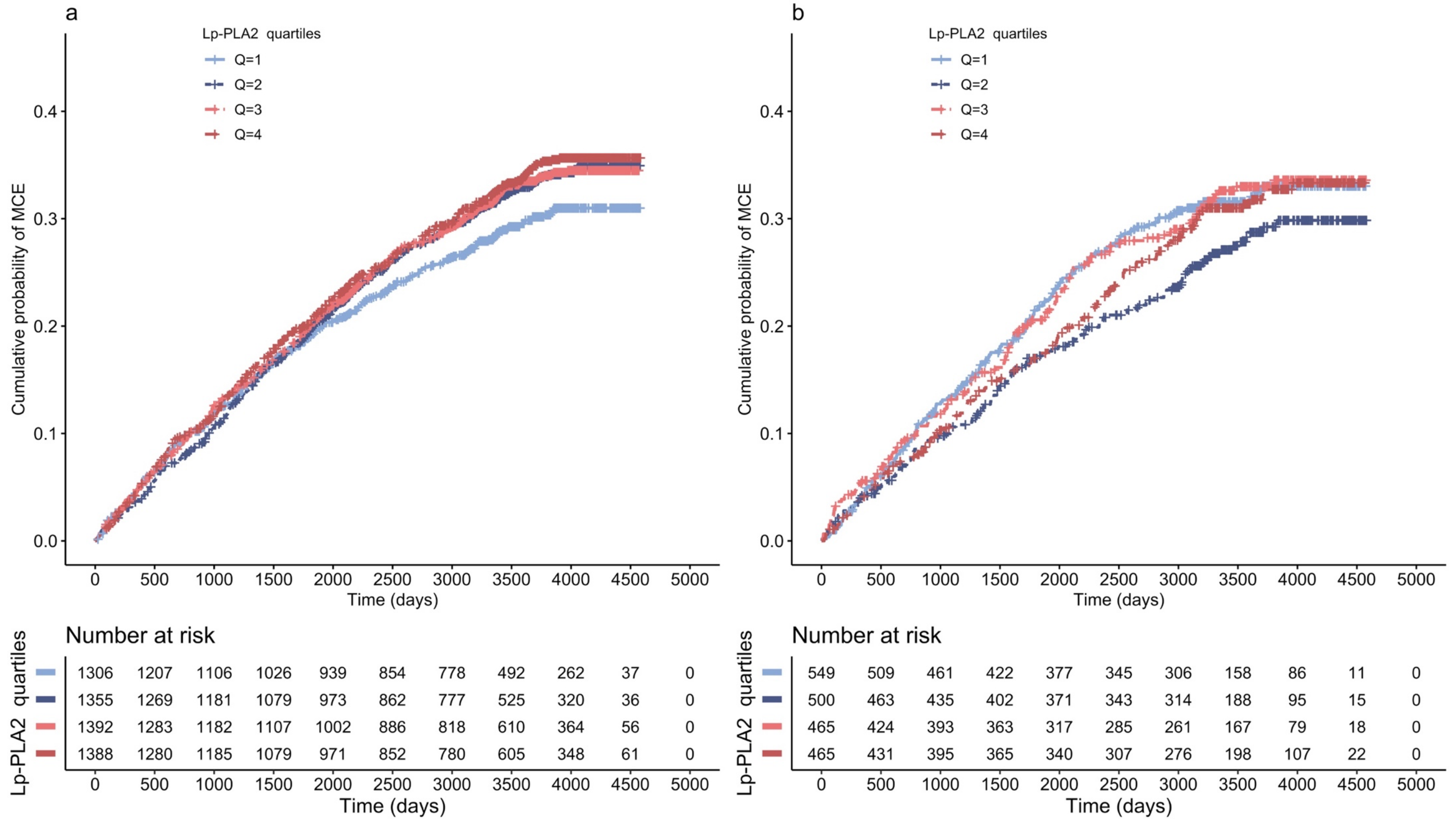

ESM Fig 3. Stratified effect of successive Lp-PLA2 quartiles on the risk of MCE by diabetes control status in GoDARTS. 1a.Cumulative hazards plot of the association in individuals with poorly-controlled diabetes. Hazard Ratio (95% Confidence Interval): Q4 v. Q1 = 1.35 (1.16, 1.57). Figure 1 b. Cumulative hazards plot of the association in individuals with well-controlled diabetes HR (95% confidence interval) Q4 v. Q1 = 1.10 (0. 80,1.40). Model presented in graphs are unadjusted for covariates. Findings from full adjusted model in ESM Table 10.

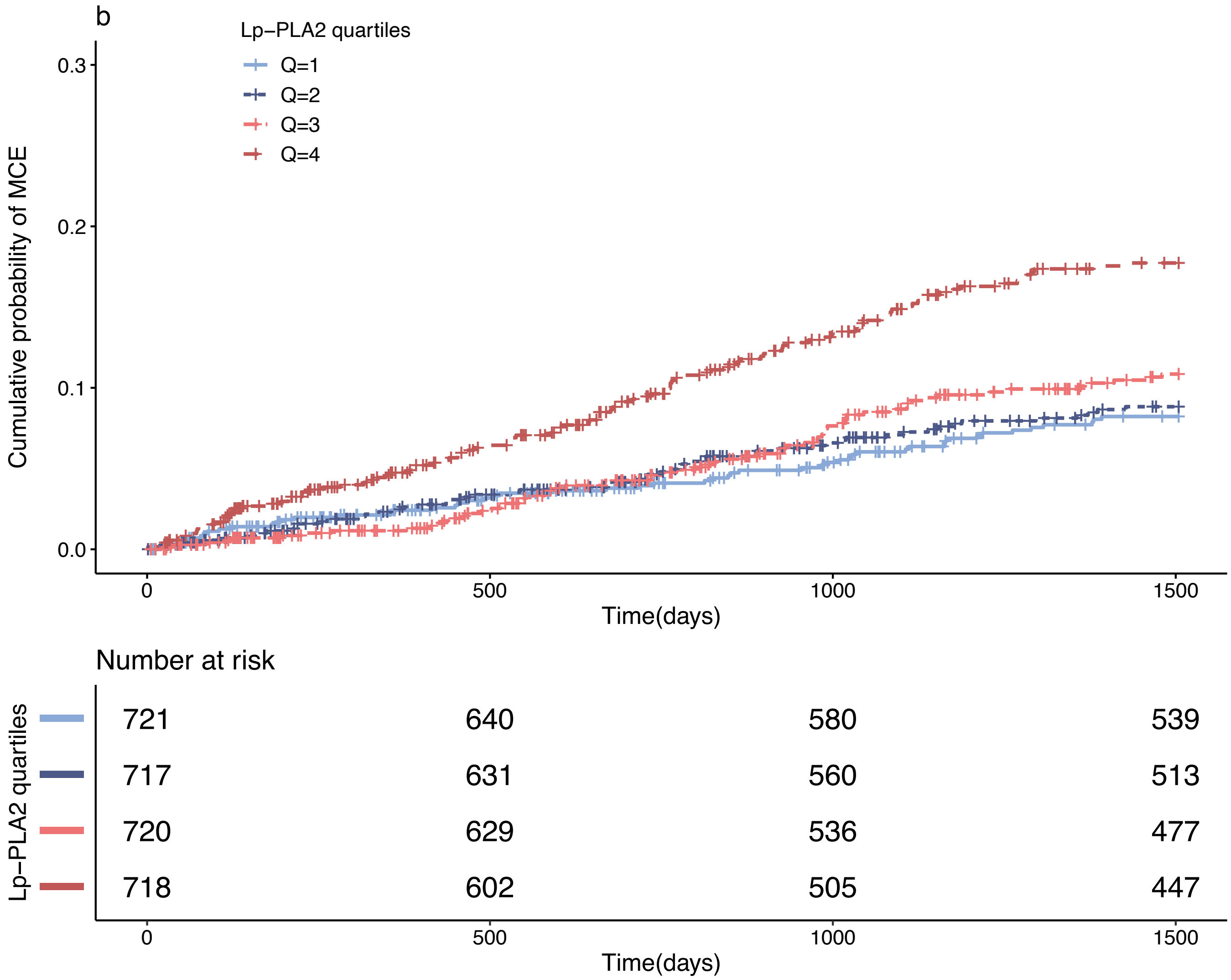

ESM Fig 4. STABILITY trial: Hazards of increasing quartiles of Lp-PLA$_2$ activity on the risk of MCE in participants with type 2 diabetes receiving placebo. This plot represents main effects. Hazard ratio (95% confidence interval) for Q4 v. Q1 = 2.50 (1.70,3.68), P <0.0001. Hazard ratio (95% confidence interval) for Q4 v. Q3-1 = 2.18 (1.62, 2.93) <0.0001. Findings from full adjusted models presented in ESM Table 11.

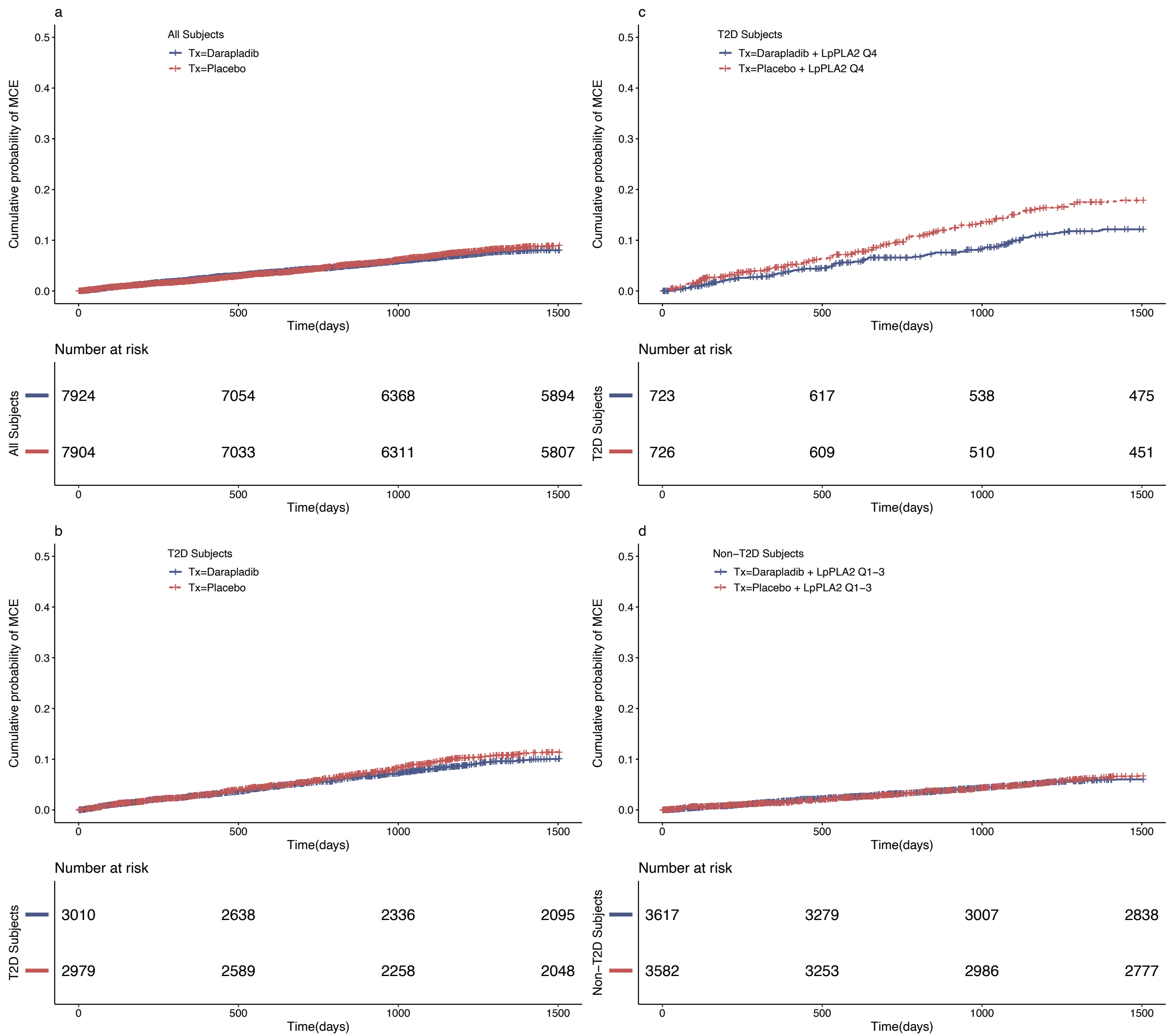

ESM Fig 5. Panels displaying the effect of darapladib therapy in different sub-groups of the STABILITY trial. 3a Effect of darapladib therapy in the entire STABILITY population HR 0.90(95%CI:0.81,1.01) P = 0.07, 3b. Effect in trial participants with type 2 diabetes 0.89(0.75,1.05) P = 0.15, 3c. Effect in the higher risk group (type 2 diabetes + Lp-PLA2 activity in Q4) and 0.64 (0.48,0.86) P= 0.003 and 3d. Effect in the lower risk group (no type 2 and Lp-PLA2 activity in Q1-3). HR 0.91, (95%CI 0.75, 1.10), P=0.34. Non-T2D, no diabetes. Models presented in graphs are unadjusted for covariates (Findings from full adjusted models in ESM Table 13).
